# Microarray analysis of gene expression induced by sexual contact in *Schistosoma mansoni*

**DOI:** 10.1186/1471-2164-8-181

**Published:** 2007-06-20

**Authors:** Michael Waisberg, Francisco P Lobo, Gustavo C Cerqueira, Liana KJ Passos, Omar S Carvalho, Glória R Franco, Najib M El-Sayed

**Affiliations:** 1 Laboratório de Genética Bioquímica, Departmento de Imunologia e Bioquímica, Instituto de Ciências Biológicas, Universidade Federal de Minas Gerais, Belo Horizonte, MG, Brazil; 2 Department of Parasite Genomics, The Institute for Genomic Research, Rockville, MD, USA; 3 Laboratório de Genética Molecular de Tripanosomatídeos, Departamento de Imunologia e Bioquímica, Instituto de Ciências Biológicas, Universidade Federal de Minas Gerais, Belo Horizonte, MG, Brazil; 4 Centro de Pesquisas René Rachou, Fundação Osvaldo Cruz, Belo Horizonte, MG, Brazil; 5 Department of Cell Biology and Molecular Genetics and Center for Bioinformatics and Computational Biology, University of Maryland, College Park, MD, USA

## Abstract

**Background:**

The parasitic trematode *Schistosoma mansoni *is one of the major causative agents of Schistosomiasis, a disease that affects approximately 200 million people, mostly in developing countries. Since much of the pathology is associated with eggs laid by the female worm, understanding the mechanisms involved in oogenesis and sexual maturation is an important step towards the discovery of new targets for effective drug therapy. It is known that the adult female worm only develops fully in the presence of a male worm and that the rates of oviposition and maturation of eggs are significantly increased by mating. In order to study gene transcripts associated with sexual maturation and oviposition, we compared the gene expression profiles of sexually mature and immature parasites using DNA microarrays.

**Results:**

For each experiment, three amplified RNA microarray hybridizations and their dye swaps were analyzed. Our results show that 265 transcripts are differentially expressed in adult females and 53 in adult males when mature and immature worms are compared. Of the genes differentially expressed, 55% are expressed at higher levels in paired females while the remaining 45% are more expressed in unpaired ones and 56.6% are expressed at higher levels in paired male worms while the remaining 43.4% are more expressed in immature parasites. Real-time RT-PCR analysis validated the microarray results. Several new maturation associated transcripts were identified. Genes that were up-regulated in single-sex females were mostly related to energy generation (i.e. carbohydrate and protein metabolism, generation of precursor metabolites and energy, cellular catabolism, and organelle organization and biogenesis) while genes that were down-regulated related to RNA metabolism, reactive oxygen species metabolism, electron transport, organelle organization and biogenesis and protein biosynthesis.

**Conclusion:**

Our results confirm previous observations related to gene expression induced by sexual maturation in female schistosome worms. They also increase the list of *S. mansoni *maturation associated transcripts considerably, therefore opening new and exciting avenues for the study of the conjugal biology and development of new drugs against schistosomes.

## Background

Schistosomiasis is an important public health problem that affects approximately 200 million people, mostly in developing countries, and that poses a risk to another 600 million [1]. Mortality attributed to schistosomiasis is estimated to be around 11,000 deaths/year with an added economic burden of 1.7 million disability adjusted life-years lost per year [2]. The disease is transmitted when parasite eggs in human feces reach fresh water and hatch into miracidia. Upon infection of an appropriate snail species, miracidia transform into sporocysts that asexually reproduce generating hundreds of thousands of cercaria. The cercaria are shed by the snail and swim until they find an appropriate vertebrate host and penetrate through its skin. The worms then undergo differentiation and migrate in the bloodstream until they reach the mesenteric veins where male and female worms pair.

Eggs from paired mature schistosomes are responsible for most of the pathology caused by *S. mansoni*. Each mature female lays on average 300 eggs per day, some of which are excreted in the feces [3]. The remaining eggs end up in the liver, intestine and other organs where they cause an inflammatory reaction, producing significant scarring which leads to a variety of symptoms depending on the organ where they lodge. For instance, eggs trapped in the bowel wall may cause bloody diarrhea, cramping, and eventually inflammatory colonic polyposis [4]. Eggs that are swept back to the hepatic portal system cause granulomatous reaction in the portal tract which can evolve to hepatoportal fibrosis and portal hypertension. More interestingly, although immature worms may lay eggs which are occasionally shaped normally, those eggs are unable to induce the formation of granulomas [5].

Male schistosomes are responsible for triggering and maintaining female maturation. In absence of the male, female worms cannot migrate against the blood flow from the portal sites in the liver to the smaller mesenteric circulation where they lay their eggs [6]. Therefore, the survival of *S. mansoni *couples and the maintenance of their complete life-cycle seem to be dependent on the existence of a permanent association between sexes. Studies by Shaw and Erasmus on praziquantel (the drug of choice for treating Schistosomiasis) have shown some evidence that the drug disrupts the reproductive system of females and that subcurative doses cause a long-lasting regression of both ovary and vitelline gland 24 hours post-treatment [7]. Popiel *et al*. (1984) have shown that there is a dose-dependent and reversible effect of oxamniquine (another drug which is used for treating *S. mansoni *infections) on the female reproductive system [8,9]. Although these effects on the reproductive system are usually reversible, these data might suggest that the reproductive system of female schistosomes could provide good targets for therapeutic agents against these parasites.

In contrast to male schistosomes that undergo normal morphological development regardless of their pairing status, females from single sex infection (single-sex females) show clear differences from those which are paired to males. Virgin females are considerably smaller than paired females [10]. When paired females are separated from males they stop laying eggs and regress to an immature state, however if they are allowed to couple they maturate again [9,11-13] showing that the maintenance of sexual maturation in the female is dependent upon the presence of the male partner. The stimulus for female maturation is independent of male sperm, species and fertilization [14-17]. In addition to the morphological modifications present in single-sex females, separation of female worms leads to a large variety of changes in the physiology of the worm, culminating in the atrophy of the ovary and vitelline gland [5]. For instance, there is a great increase in DNA synthesis in single-sex females upon pairing with males. Also, paired females utilize more glucose than unpaired ones [18]. In spite of their immaturity, single-sex females reach a limited level of development, allowing them to lay eggs, which are usually malformed and non-viable [5,14]. The rates of oviposition and maturation of eggs are significantly increased by pairing of the single-sex female worms with males [15].

Different factors have been proposed to be involved in *S. mansoni *sexual maturation including physical/tactile contact [11,14,15], nutrition [6,19-21] and chemical stimuli [16,22,23]. In support to the importance of tactile contact, Michaels showed that when halves of worms (i.e. one of the two segments from worms that were cut in two halves) are allowed to mate *in vitro*, like halves (either cranial or caudal halves) always mate normally while unlike halves almost always mate abnormally, postulating that both sexes of worms have linear receptors which are used to determine dating and mating position [15]. In support to the role of nutrition, *S. mansoni *female worms from single-sex infections have limited pharyngeal musculature, thin intestinal cecal walls, lack of digestive enzymes and reduced intake of red blood cells than females from paired infections [20]. LoVerde and colleagues have suggested that the muscularity of the male worm allows it to assist the weaker females with pumping host blood into their intestine by a massaging effect of the muscular walls of the gynecophoric canal (i.e. a ventral longitudinal groove in the surface of male Schistosomatidae) [6]. Also, Cornford and Huot demonstrated that there is transfer of ^14^C glucose from male worms to females, implying that male schistosomes indeed help feed female worms [21]. Chemical stimuli such as acetone or ether extracts of male worms have been shown to induce female development *in vitro *[16]. Also, an observed cholesterol transfer from males to females may suggest a role for hormonal factors [23].

On the male parasite side, the effects of sexual pairing on male worms are very subtle and therefore have received little attention in comparison to female worms. Nevertheless, some studies show that male *S. mansoni *and *S. haematobium *worms from single-sex infections are significantly smaller and have a reduction in the number of testes when compared to worms from homologous paired infections [24,25]. Using the data published by Basch and Gupta it is possible to observe that pairing also stimulates a higher rate of cell division (as measured by the number of nuclei per section unit area) in males when bisexually paired males and unisexual unpaired males are compared [26]. The stimulatory effect of female schistosomes on male worms has also being studied at the biochemical and molecular level. For instance, worms from single-sex infections have more glutathione than worms from paired-infections and this difference can be reversed by incubation of those worms with females [27,28]. Also, female worms stimulate tyrosine incorporation, lipid accumulation and lipase utilization in males [23,29]. Another interesting fact is that paired male schistosomes express a 79-KDa protein in its gynecophoral canal and that this protein detection is severely limited at the surface of single-sex male worms [30]. This protein, which shows wide distribution on the surface of adult female worms has homology to developmentally-regulated homotypic adhesion molecules and is suspected to be essential for continued interaction between sexes and sexual development of schistosomes.

Several expression analyses were recently performed in schistosomes using microarrays to study gender differences in gene expression of *S. mansoni *and *S. japonicum*, as well as stage specific, strain specific, maturation specific, and species specific differences [31-38]. Microarrays were also used to investigate the vertebrate hepatic host response to infection with *S. mansoni *[39]. These studies have provided important information concerning the biology of the parasite and the host response. In this study, we describe the use of DNA microarrays to study the effect of sexual pairing of *S. mansoni *(Brazilian LE Strain) in the adult gene expression. To accomplish this objective we compared worms from single-sex infections with worms from paired-infections. Our results reveal novel information of genes putatively involved in oogenesis and sexual maturation of schistosomes and allow the identificaton of new possible targets for therapeutic intervention.

## Results and discussion

### Evaluation of replication quality

In order to investigate the quality of our replicates we tested the correlation between different types of replicates using LIMMA (Linear Models for Microarray Data) [40]. To generate single-sex worms we exposed multiple *Biomphalaria glabrata *snails each to a single miracidium and harvested the cercariae shed by them after one month. Multiple mice were subsequently infected with either single-sex or mixed-sex cercariae and the worms harvested 51 days post-infection. The experimental design included three types of replicates: biological, technical and in-slide replicates encompassing 6 slides in total for each experiment. In brief, single-sex and paired-infection worms were harvested from multiple mice and pooled separately in pools of 500 worms or more (each pool contained worms from different mice). The RNA from each pool was extracted separately and then hybridized to the slides. Three biological replicates were used for each experiment and each of them was hybridized twice (dye swap). Because each probe was printed in duplicate we computed also the correlation between in-slide replicates.

The approach used was to estimate a common correlation for all the genes within blocks corresponding to each type of replicate. As would be desired, the level of correlation between in slide technical replicates was between 0.90 and 0.92 for male and female experiments respectively. Dye swap replicates displayed a correlation between -0.85 (male experiment) to -0.89 (female experiment), indicating that the fluorescent dyes introduce a very minimal bias in hybridization intensities. The correlation between our biological replicates (using RNA isolated from different pools of worms) was 0.73 for the female experiment and 0.61 for the one investigating sexual pairing on male worms. Our results are in agreement with the data published by Hoffmann and colleagues who found an in-slide replicate correlation of 0.91 and a correlation ranging from 0.81 to 0.90 between slides (biological) [31,41].

### Differentially expressed genes

**Figure 1:**
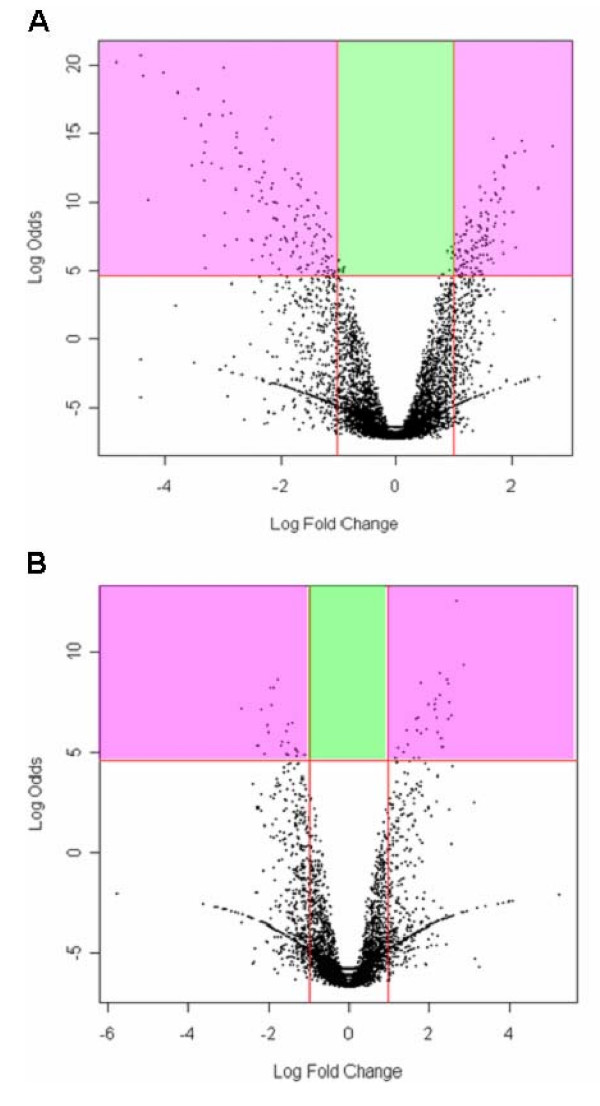
***The volcano plot for the female (A) and male (B) datasets***. Volcano plot identifying genes which are significantly different between mature and immature female (A) and male (B) *S. mansoni *adult worms. The plot displays print-tip normalized fluorescence intensity ratios for all replicates on a two-axis system. The x-axis corresponds to the log_2 _of the fold change between mature and immature worms and the y-axis corresponds to the Log Odds (or B value) which is the odds (or probability) that a certain gene is differentially expressed. A Log Odds value of 0 (horizontal line in each graph) corresponds to a 50-50 chance that the gene is differentially expressed. The higher the Log Odds for each gene, the higher the probability that the gene is differentially expressed and not a false positive. The pink areas show genes that were considered as differentially expressed, i.e. it showed a fold difference greater than or equal to 2 (log_2 _fold change ≥ 1 or ≤ -1) and logOdds ≥ 4.6 (99% of chance of being statistically significant). The green area shows genes that have logOdds within the acceptable range but which have fold differences smaller than 2.

Statistical analysis of the data using a linear model to assess differential expressed genes revealed a large number of transcripts of gene products putatively related to sexual maturation in both male and female schistosomes (Table 1 and 2). Only genes with at least two-fold difference (log_2 _ratios ≥ 1 or ≤ -1) and logOdds ≥ 4.6 were considered differentially expressed. Volcano plots, where the magnitude of the gene expression ratio is displayed on the x-axis and the significance of the difference in expression between groups on the y-axis, are shown in (Figure 1). 265 genes were observed to be differentially expressed between mature and immature female parasites and 53 genes between mature and immature male worms.

**Table 1:** Top 30 most differentially expressed genes in adult female schistosomes.

**Name**	**M**	**A**	**P Value**	**B**
Similar to putative ATPase N2B (HFN2B) 5E-05 possible antisense	-4.41895	10.84994	1.35E-09	20.60678
Extracellular superoxide dismutase [CU-ZN] precursos (EC1.15.1.1) (EC-SOD) (6E-97)	-4.83772	11.60992	1.35E-09	20.10658
Unknown 2572 possible antisense	-2.97935	9.425281	1.35E-09	19.78673
Similar to female specific eggshell protein ORF 2 (1E-144)	-4.00956	10.33001	1.57E-09	19.40354
Unknown 1477	-4.37742	9.800644	1.70E-09	19.13286
Unknown 535 possible antisense	-3.40857	8.462215	4.12E-09	18.16237
Unknown 1495	-3.78529	7.836334	4.60E-09	17.92087
Unknown 926	-2.97584	9.805078	8.07E-09	17.27377
Unknown 778	-2.84958	9.831668	1.72E-08	16.36883
Unknown 2609 possible antisense	-3.23019	9.170634	1.72E-08	16.34812
Unknown 1805 possible antisense	-2.99634	7.133567	1.74E-08	16.24925
*S. mansoni *18S rRNA gene, complete sequence	-2.15136	9.47819	1.87E-08	16.09624
*S. mansoni *eggshell protein mRNA, 3' end	-3.65217	8.873222	1.94E-08	15.98481
Similar to *S. mansoni *small subunit rRNA gene (DNA level 0.0)	-3.35601	12.32288	2.74E-08	15.58348
Unknown 3085	-2.23101	10.75529	3.35E-08	15.32323
Unknown 46	-2.74433	7.536724	4.14E-08	15.05545
*S. mansoni *ribosomal intergenic spacer DNA	-2.74253	10.10216	5.38E-08	14.74033
Unknown 398	1.685296	12.69879	6.12E-08	14.54298
*S. mansoni *small subunit rRNA gene	-2.12156	13.00096	6.12E-08	14.50706
Similar to plasma kallikrein precursor (EC 3.4.21.34) 1E-11	2.181398	12.00887	6.63E-08	14.37559
Unknown 475	-3.28488	8.766573	6.63E-08	14.33036
Similar to aldehyde dehydrogenase 1 family, member B1 (1E-160)	2.708917	13.41307	8.44E-08	14.04777
Unknown 4495	-2.75499	7.45355	9.68E-08	13.86973
Unknown 260 possible antisense	2.245423	13.61931	1.18E-07	13.6361
Unknown 2268	-2.67757	7.592068	1.25E-07	13.51174
Similar to RAS-like GTP-binding protein Rho (2E-89)	2.059329	12.85641	1.25E-07	13.46496
Unknown 3554	-3.30468	11.33136	1.25E-07	13.45976
Unknown 2741	1.934138	11.25831	1.52E-07	13.22701
*S. mansoni *28S rRNA gene gap region	-2.27931	8.995688	2.01E-07	12.91806
Similar to eggshell protein precursor (chorion protein) 2E-12 possible antisense	-3.34778	10.20964	2.04E-07	12.84231

Top 30 most differentially expressed genes in adult female schistosomes. M values are the log-ratio of the two expression intensities. A values are the mean log-expression of the two. Genes with negative M values are down-regulated in immature females (or up in paired-infection worms) and genes with positive M values are up-regulated in immature worms.

**Table 2:** Top 30 most differentially expressed genes in adult male schistosomes.

**Name**	**M**	**A**	**P Value**	**B**
*S. mansoni *female specific polypeptide mRNA	-2.66936	12.21286	4.91E-06	12.51864
Similar to putative ATPase N2B (HFN2B) 5E-05 possible antisense	-2.8511	10.52274	6.99E-05	9.333646
*S. mansoni *small subunit rRNA gene	-2.27115	11.68601	6.99E-05	8.885995
Unknown 4206	1.760138	12.02897	6.99E-05	8.616023
Similar to *S. mansoni *small subunit rRNA gene (DNA level 0.0)	-2.45433	9.02915	6.99E-05	8.605466
Unknown 3242 possible antisense	-1.80035	11.01702	6.99E-05	8.465827
Similar to rat snRNP-associated polypeptide N (8E-33)	-2.46543	11.86099	6.99E-05	8.424534
*S. mansoni *glucose transporter protein (SGTP2) mRNA, complete CDS	1.864996	11.93532	6.99E-05	8.221553
Similar to actin, cytoplasmic 3 (beta-actin 3) 9E-79	1.933797	11.65976	6.99E-05	8.206634
*S. mansoni *5.8S ribosomal RNA and 28S ribosomal RNA genes, partial sequence, and internal transcribed spacer, complete sequence	-2.28119	10.0614	9.51E-05	7.819557
Unknown 3085	-2.18385	9.768994	0.000105	7.632409
Similar to VKG protein (4E-08) and collagen alpha I(IV) chain precursor (8E-08)	-2.50168	8.788424	0.00011	7.456866
Unknown 926	-1.98422	8.257669	0.00011	7.378939
Unknown 1783 possible antisense	-2.15972	11.35038	0.00011	7.344978
Similar to Ras-like GTP-binding protein Rho (2E-89)	1.921029	10.26005	0.00011	7.295446
Unknown 43	2.663762	12.76366	0.000112	7.151475
Unknown 4470	2.191225	8.598522	0.000112	7.122331
*S. mansoni *internal transcribed spacer 2, partial sequence	-2.14823	10.26113	0.000112	7.105776
*S. mansoni *heat shock protein 86 mRNA, complete CDS	-2.5539	12.64794	0.000147	6.767502
Similar to splicing factor 3A subunit 2 (spliceosome associated protein 62) 1E-15 possible antisense	-1.73796	10.33214	0.000147	6.674734
Similar to putative sRNP (7E-07)	-2.21174	9.511203	0.000147	6.664802
Unknown 2858	-2.47374	7.487032	0.000147	6.65119
Similar to NADH dehydrogenase 3 (NADH dehydrogenase subunit 3) 6E-35 possible antisense	-1.66681	10.67992	0.000147	6.616512
Unknown 70	1.387855	13.84221	0.000173	6.419602
*S. mansoni *cathepsin B (SM31) mRNA, complete CDS	1.506688	12.30752	0.000173	6.379095
Unknown 4275	2.032754	9.699271	0.000182	6.290137
Unknown 971	-1.92642	13.63907	0.000196	6.153524
Unknown 1927 possible antisense	-1.69877	11.13445	0.000196	6.122623
Unknown 312 possible antisense	-1.43402	12.21602	0.000196	6.115432
Unknown 3940	1.553483	13.10834	0.000199	6.070285

Top 30 most differentially expressed genes in adult male schistosomes. M values are the log-ratio of the two expression intensities. A values are the mean log-expression of the two. Genes with negative M values are down-regulated in immature females (or up in paired-infection worms) and genes with positive M values are up-regulated in immature worms.

Our results show that 55% of the genes which are differentially expressed are expressed at higher levels in paired females while the remaining 45% are more abundant in immature female worms. The differentially expressed transcripts (Table 1) in female worms included many previously identified gender related genes including the p14 eggshell protein gene (Contig 1625, SCMESP), other egg shell proteins (Contig 6116, Contig 1557, SMEGGPRO, Contig 867), extracellular superoxide dismutase (Contig 119, SCMSOD2, SEG_SCMOD, SCMSOD3, SCMSODM), tyrosinase (Contig 948, Contig 1617), cathepsin B (SMA312106), dynein light chain (SMU55992), gynecophoral canal protein (SMU47862), aspartate aminotransferase (Contig 1636), diacylglycerol acetyltransferase (Contig1301) and female specific protein fs800 (SCMFS800) [41-43].

Fs800, has already been shown to be up-regulated in the vitelline cells during maturation of female worms and has been linked to the production and maintenance of eggs [42]. The gynecophoral canal protein (SMU47862), also previously shown to be differentially expressed between male and females, has been implicated in male/female communication or interaction [44]. Our data showing down regulation of the small G protein Ras in female is also consistent with published observations [45]. Tyrosinase, extracellular superoxide dismutase, glucose transporter protein I, diacylglycerol acetyltransferase 2-like protein, aspartate aminotransferase and egg shell proteins are known to be differentially expressed between male and females suggesting that they might be related either to sexual determination, maturation, reproduction or other sex specific activities [31,41]. Our results show that these genes, in addition to being differentially expressed between genders, are also differentially expressed in female worms in different sexual maturation stages. A great number of transcripts not previously associated with sexual maturation were also observed. These included membrane antigens, importin beta-1, *S. mansoni *glucose transporter, myosin heavy chain, rho2 gtpase, lecithin-cholesterol acytransferase, apoferritin-2, various ribosomal proteins and others (Table 1). Of the 265 genes we identified as differentially expressed in females, 142 did not contain any annotation because they did not show significant similarity with sequences contained in the NCBI at the time the array was printed [41]. These genes represent a multitude of unexplored proteins which might be potential targets for new therapeutic agents.

Of the genes with an associated gene ontology, genes that were up-regulated in single-sex females were mostly related to energy generation (i.e. carbohydrate and protein metabolism, generation of precursor metabolites and energy, cellular catabolism, and organelle organization and biogenesis) while genes that were down-regulated were related to RNA metabolism, reactive oxygen species metabolism, electron transport, organelle organization and biogenesis and protein biosynthesis (Figure 3). The increased amount of antioxidants in mature female is in agreement with their increased need to detoxify hemoglobin byproducts associated with increased blood cell consumption [46]. On the other hand, one would expect that paired-infection females express more carbohydrate and protein metabolism genes than single-sex worms. The fact that we found more of those genes in single-sex females supports Basch's nutritional theory of sexual maturation. If males do indeed help females to feed, this could allow mature worms to direct their metabolism to the more important egg generation (since the male is already taking care of energy generation) and therefore genes related to energy generation should be decreased in mature females in relation to immature. Among the genes that are down-regulated there are six genes coding for eggshell proteins. This is expected since paired females are egg-laying machines capable of converting the equivalent of their own body dry weight into eggs [47] and consuming about 8 millions erythrocytes per day. In light of this high metabolic activity of mature females, it has been proposed that female-specific gene products associated with the metabolic machinery of egg production and hemoglobin catabolism should be highly represented in molecular biological studies of gender [47] which is the case in this study. The fact that genes associated with organelle organization were both up and down regulated in immature females could possibly be explained by differences in vitelline cells production among mature and immature females. It has been shown that paired female schistosomes produce several thousands vitteline cells per day [48]. This high mitotic activity must demand high amounts of protein synthesis and the participation of an array of proteins related to organelle biogenesis and organization. The fact that some genes related to this process were up-regulated in immature females is more difficult to explain but it could be due to some compensatory mechanism whereby immature females make up for the lack of nutritional support by males by producing new cells.

For male parasites, our results show that 56.6% of the differentially expressed genes were present in larger quantities in mature males while the remaining 43.4% are more expressed in males from single-sex infections. The fact that more genes were differentially expressed between females than between males is consistent with the fact that immature males present less morphological and biochemical differences than immature females. Nevertheless this data does not agree with a previous study of gene expression induced by sexual maturation which found a similar number of differentially expressed genes in males and females [36]. This difference might have been caused by differences in the experimental design, data analysis parameters, host characteristics or differences in the parasite strain used. Indeed, results from Moertel and colleagues have shown that more than 600 genes are differentially expressed when different strains *S. japonicum *are compared under identical conditions [38]. When the gene lists of our experiments and Fitzpatrick and Hoffmann [36] are compared (See Additional file 1) we find less than 10% of genes which are common to the two datasets and in one case (single-sex males) we did not find any genes in common. Our data also shows that mature male worms express more genes related to the metabolism of proteins, nucleotides, macromolecules, ion transport, and metabolite/energy generation than unpaired worms, which suggests that paired worms are more metabolically active. The data agrees with Fitzpatrick and Hoffmann (2006) in that more genes potentially involved in RNA metabolism were differentially expressed between mature and immature male worms [36], nevertheless we did not find a greater proportion of genes in the structural molecule activity GO category.

Interestingly, the data from Khalil and Mansour show that male worms from single-sex infections are in fact smaller than worms from paired-infections [24]. Although the authors do not explain this phenomenon, our results suggest that the difference in size could be linked to the higher expression of genes involved in energy generation and accumulation (Figure 3). The fact that paired male worms absorb and phosphorylate glucose more rapidly than unpaired worms also reinforces this hypothesis [18]. Nevertheless, we observed a paradoxical higher expression of Cathepsin B Sm31 in unpaired male worms. Gotz and Klinkert demonstrated that this protein is an enzyme which is involved in the degradation of hemoglobin in the digestive tract of schistosomes [49].

Another interesting observation is that unpaired male worms synthesize more lecithin-cholesterol acyltransferase (LCAT), acyl-CoA binding proteins (ACPB) and fatty acid binding protein (FABP). In humans LCAT is responsible for the formation of cholesteryl esters starting from lecithin and cholesterol and it is the LCAT on the surface of nascent HDL particles that convert cholesterol and phosphatidylcholine of chylomicron and VDLD in cholesteryl esters. The accumulation of cholesteryl esters in the nascent HDL convert it in mature HDL and directs it back to the liver where the cholesterol is usually unloaded. The acyl-CoA binding proteins are small proteins which bind to long and medium chain acyl-CoA esters with high affinity and which can act as intracellular carriers of acyl-CoA esters. FABPs, are a members of a family of cytosolic lipid binding proteins and that have been investigated as possible vaccine candidates [50]. Furthermore, Gobert and colleagues demonstrated that the *S. japonicum *FAPB is confined to lipid droplets in male worms that are probably involved with the nurture of female worms [51].

In studies with humans and marmosets (*Callithrix jacchus*) the plasmatic levels of total cholesterol, esterified cholesterol, triglycerides and phospholipids were significantly reduced after infection with *S. mansoni *[52,53]. Similar effect was observed by Doenhoff and colleagues, who reported a reduction in atherosclerosis in rats predisposed to cardiovascular disease (apoE^-/-^) after infection with *S. mansoni *[54]. They suggested that this reduction could be due to a modulatory effect of the infection by *S. mansoni *on lipid metabolism in the host. On the other hand, Ramos and colleagues proposed that the cholesterol reduction could be caused by changes in the LCAT and/or ACAT (acyl-CoA-cholesterol acyl transferase) activity, synthesis or secretion produced by the liver of infected animals [52]. Taking into consideration that schistosomes are not capable of synthesizing cholesterol [55,56], the fact that sexual pairing has an effect upon so many genes related to fatty acid and cholesterol metabolism in male worms becomes even more relevant.

### Validation of microarray results using real time quantitative PCR

In order to validate the microarray results we performed real-time RT PCR. Contigs considered to be differentially expressed were first checked against the *S. mansoni *genome to evaluate the validity of the probes printed on the arrays and to remove redundant contigs (i.e. different contigs which represented the same gene).

Primer pairs were designed for all genes considered as differentially expressed and then tested against the genome using electronic PCR [57] and BLAST [58] to select only those that amplified a unique region of the genome. 41 female and 20 male primer pairs qualified, of which approximately 20 of each were randomly selected for real time quantitative PCR. The results from the quantitative PCR for the male and female experiments were compared against the results of our microarray hybridizations using linear regression analysis [59] and showed a strong correlation (R = 0.84 and R = 0.67 respectively).

## Conclusion

The results presented on this article confirm previous results related to gene expression induced by sexual maturation in schistosome worms. It also contributes a wealth of information about genes (either characterized or unannotated) that may be involved in this process. Since most of the differentially expressed genes do not have homology to other genes, further work is necessary to characterize these transcripts and assign biological functions to them. Once the *Schistosoma mansoni *genome is fully annotated, it will be possible to extract more interesting information from our data. Nevertheless, we believe that the myriad of up- and down- regulated genes we describe here open new and exciting doors for the study of the conjugal biology and the development of new drugs against schistosomes.

## Methods

### Unisexual infections

*S. mansoni *lifecycle was maintained at Instituto de Pesquisa Rene Rachou, Fiocruz (Brazil). *S. mansoni *(LE Brazilian strain) is maintained in *Biomphalaria glabrata *as intermediate host. Outbred Swiss mice were used as definite hosts. To obtain single-sex adult worms, *B. glabrata *snails were exposed to a single miracium, which was generated from eggs obtained from the liver of infected mice [60]. After one month the snails were tested for positive infection by verifying the shedding of cercaria following exposure of the snails to artificial light [61]. 100–200 cercaria from each snail were injected in the peritoneum of female Swiss mice.

Mice were housed conventionally in polypropylene cages with stainless steel screen covers. All animals received lab mouse chow and water *ad libitum*. The animals were sacrificed at 51 days post-infection according to ethical procedures and adult worms were obtained by portal perfusion [62]. The worms were washed in cold saline solution and checked by microscopy for the presence of possible undesirable mixed-sex infections. We separated the single-sex adult worms in multiple pools (each one containing >500 worms which were originated from multiple mice) which were frozen at -80°C until further processing of the samples.

### Paired-infections

To obtain worms from paired-infections, *B. glabrata *snails were exposed to multiple miracidia and the cercaria from these snails used to infect Swiss mice. Each mouse received approximately 100 cercaria and the animals were sacrificed at 51 days post-infection. The perfusion was performed in the same fashion as for single-sex worms. The worms were washed with cold saline solution and carefully separated by their sex with forceps under the microscope. Worms from each sex were pooled separately (>500 worms/pool) and frozen until further processing.

### RNA extraction and amplification

Total RNA was extracted using Trizol reagent (Invitrogen Life Technologies, CAT#15596-026) according to the manufacture's instructions. The RNA was quantified using a Nanodrop ND-100 UV/Vis spectrophotometer (NanoDrop Technologies, USA) and the overall RNA quality was assessed using denaturing gel electrophoresis [63]. Two μg of total RNA from each sample were amplified by doing two rounds of linear amplification using the Amino Allyl MessageAmp II kit (Ambion, CAT#1753). The amplification was done according to manufacture's specifications and aaUTP was used on the second round of amplification so that the final product could be labeled using indirect labeling.

### RNA labeling and hybridization

Aminoallyl amplified RNA was labeled using Cy3 and Cy5 by indirect labeling according to a modified version of the TIGR's standard operational procedure [64]. In brief, for each hybridization 15 μg of amplified RNA was dried in a speed-vac, resuspended in pH 9.0 carbonate buffer and incubated for 5 minutes at RT. After this incubation, 4.5 μL of one of the two dyes dissolved in DMSO (Amersham, CAT#PA23001 and PA25001) was added to the solution and the samples were incubated at RT in the dark for one hour. Labeled RNA was purified away from unincorporated dye using RNeasy MinElute Cleanup Kit (Quiagen, CAT#74204). The Cy3 and Cy5 labeled samples were then combined, dried again, and resuspended in hybridization buffer (50% formamide, 5× SSC, 0.1% SDS).

### Microarray hybridization and experimental design

The pools of female or male worm RNA from single-sex infections were hybridized against RNA from paired-infections from worms of the same sex. Each experiment was performed in duplicate (technical replicate) using a dye swap design in order to account for dye biases. The samples were hybridized using oligonucleotide DNA microarrays obtained from the laboratory of Karl Hoffmann at the University of Cambridge, UK. The arrays contained 7335 oligonucleotides (50 mers) spotted in duplicate. Each oligonucleotide represented either a singleton (a single EST sequence) or a contig (contigous sequences of overlapping EST sequences) generated by assembly with CAP3. The oligonucleotides were designed based on transcriptome information available at the time [41]. Briefly, the slides were pre-hybridized by placing them in coupling jars containing pre-hybridization solution (5× SSC, 0.1% SDS, 1%BSA) for 40 minutes at 42°C. The slides were then washed by dipping 10 times in a beaker containing DI water, the water was changed and the operation repeated once. The slides were spun dry using a table-top high speed microarray centrifuge (TeleChem International Inc., USA). The samples (resuspended in hybridization) were hybridized overnight under cover slips inside Corning^® ^hybridization chambers (Corning, USA) which were kept in a water bath at 42°C in the dark. Slides were washed two times for five minutes each in low stringency wash at 42°C (2× SSC, 0.1% N-lauroysarcosine), followed by two washes for 5 min in medium stringency wash (0.1× SSC, 0.1% N-lauroysarcosine) at RT and 2 washes for 5 minutes each in high stringency wash solution (0.1× SSC). Slides were spun dry and scanned using a microarray dual channel laser scanner (GenePix 4000B, Molecular Devices, USA) at 10 μm resolution, 100% laser power and PMT levels which were adjusted in order to obtain similar distributions of red and green signal intensities.

### Real time RT-PCR

A subset of genes predicted to be differentially expressed was selected for validation using real-time RT-PCR. Total amplified RNA (2 μg) from mixed sex infection and single sex infection was used for reverse transcription using TaqMan Reverse Transcription Reagents (Applied Biosystems; Cat# N808-0234). Products were amplified using the Applied SYBR Green Masters Mix kit (Applied Biosystems, Cat#4309155) in an ABI Prism 7900HT Sequence Detection (Applied Biosystems) with the following profile: 50°C for 2 min, 95°C for 10 min; 40 cycles of 95°C for 15 s and 60°C for 1 min. Each reaction was performed using 1 μL of cDNA from the RT reaction using a final volume of 20 μL (PCR Master Mix 1×, 200 nM of each primer). Expression levels of *S. mansoni *alpha-tubulin (accession number M80214) were used as endogenous control within each sample. Relative levels of gene expression were calculated using the 2^-ΔΔCT ^method [65]. Each sample was analyzed for primer dimer, contamination or mispriming by inspection of their dissociation curves.

### Data analysis

Overall, analysis of sexual pairing induced gene expression encompassed 6 slides (for each experiment), incorporating 3 replicates and dye swaps. The data, which are MIAME compliant [66], were submitted to ArrayExpress at EBI using MIAMExpress [67]. Spots were analyzed using GenePix Pro and flagged according to their quality (i.e. spots were flagged as 'Bad' whenever they were contaminated with particles, were smeared or dilated, had irregular shape or were in areas of high background; otherwise they were flagged as 'Good'). Raw intensity data were analyzed using the R statistical language [68]. The data were inspected for spatial biases on both the red and green channel (background and signal), for print-tip bias, dye bias and intensity dependent bias using the maArray package [69]. The data were then print-tip normalized using a modified version of the robust spline method [40,70]. The statistical analysis was performed using a linear model incorporating replication information. The in-slide, dye swap and pool's replicate correlations were calculated using the duplicate Correlation function of LIMMA [71]. A list of differentially expressed genes was generated by applying a Bayesian smoothing to the linear model fit. Genes that had log odds > 4.6 (99% probability that the gene is differentially expressed between the conditions being compared) and M values >1 or <-1 (2 fold difference among groups) were considered as significant. Because the slides were designed based on the assembly of ESTs from *S. mansoni *and because the genome wasn't available at the time the slides were printed, we decided to test the differentially expressed genes against the complete genome. In brief, pairs of primers were designed for all differentially expressed genes (M values <1 or <-1 and logOdds > 4.6) using a web tool (Genscript Corp, USA [72]) and then tested against the genome and the predicted complete CDSs using mePCR [45] in order to see if each primer pair would theoretically amplify a single region of the genome. Primer pairs that amplified more than one region were discarded. We further tested the primers to see if two primer pairs would amplify the same gene (predicted CDS) in order to remove redundant genes (i.e. genes that were spotted more than one time at the arrays because of assembly problems).

Further analysis was performed using Blast2GO [73]. This software allows the evaluation of differences in annotation between two groups of data. The analysis of GO terms association for the genes considered differentially expressed in both experimental groups, for the two experiments, was performed by using the *combined graphs *function of the software. These graphs allow the visualization of the combined annotation of a group of sequences and this can be used to study the biological meaning of a subgroup of sequences. Combined graphs are a good alternative to *enrichment analysis *because they don't require a reference group and allow the use of small numbers of sequences [74].

## Abbreviations

ACAT – Acyl-CoA-cholesterol acyl transferase

ACBP – Acyl-CoA Binding Protein

BSA – Bovine Serum Albumin

CDS – Coding Sequence

DALY – Disability Adjusted Life Years, the sum of years of potential life lost due to premature mortality and the years of productive life lost due to disability.

EBI – European Bioinformatics Institute

DMSO – Dimethyl sulfoxide

DNA – Deoxyribonucleic acid

EST – Expressed Sequence Tags

FABP – Fatty acid binding protein

GO – Gene Ontology

HDL – High Density Lipoprotein

LCAT – Lecithin-Cholesterol Acyltransferase

LDL – Low Density Lipoprotein

LIMMA – Linear Models for Microarray Data

MIAME – Minimum Information About a Microarray Experiment

PCR – Polymerase Chain Reaction

RNA – Ribonucleic acid

RT – Reverse Transcription

SSC – Sodium Chloride Sodium Citrate

VLDL – Very Low Density Lipoprotein

WHO – World Health Organization

## Authors' contributions

MW led the design of the study, performed the RNA amplifications, hybridizations, real time PCRs, statistical analysis of the data and drafted the manuscript. LKJP carried out the unisexual and mixed infections of *B. glabrata *and injection of mice. FPL carried out the mouse perfusions, separation of worms and RNA extractions. GCC participated in the data analysis performing the mePCR, real time PCR oligonucleotides design and subsequent data treatment. GF, NES and OSC participated in the design and coordination of the study and helped to draft the manuscript. All authors read and approved the final manuscript

**Figure 2:**
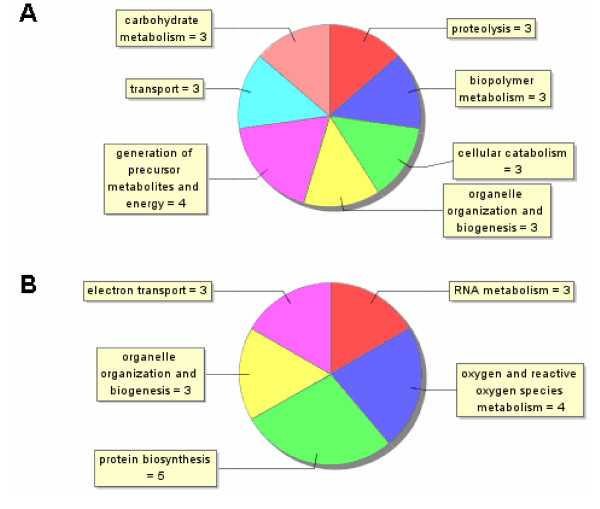
***Distribution of Gene Ontology terms for the differentially expressed genes in female worms***. A. Pie plot showing the Gene Ontology classification for the genes that were up-regulated in adult single-sex female worms. The graph does not contain all genes that were up-regulated since the majority of those do not have assigned GOs. B. pie plot showing the distribution of GOs for the genes that were down-regulated in adult single-sex female worms. The graph does not contain all genes that were down-regulated since the majority of those do not have assigned GOs.

**Figure 3:**
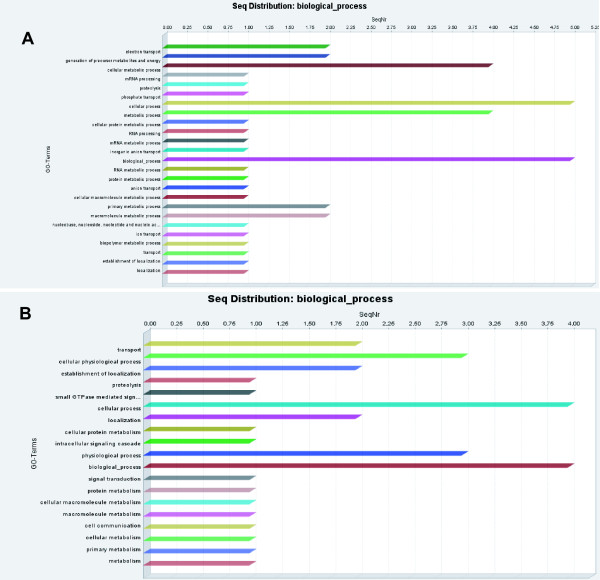
***Distribution of Gene Ontology terms for the differentially expressed genes in male worms***. Bar plot representing the Gene Ontology term distribution for the genes more expressed in: A. paired adult male *S. mansoni*; B. unpaired adult male *S. mansoni*.

## Supplementary Material

Additional File 1 Comparison of our dataset and Fitzpatrick and colleagues (2007) dataset. Click here for file
