# Supplementary material for: Microarray analysis of gene expression induced by sexual contact in Schistosoma mansoni
Source: BMC Genomics. 2007 Jun 20;8:181. doi: 10.1186/1471-2164-8-181 (PMC1929073; doi:10.1186/1471-2164-8-181)
Supplement: Additional File 1 — Comparison of our dataset and Fitzpatrick and colleagues (2007) dataset. [file 1471-2164-8-181-S1.pdf]

Table – Gene Ontologies of the genes found in the intersection of datasets

	Biological process	Molecular Function	Cell component
Paired females			integral to membrane
		DNA binding	nucleus
	amino acid metabolism<br>biosynthesis	aspartate transaminase activity<br>transaminase activity<br>transferase activity	
	eggshell formation		
	transcription from Pol I promoter	protein binding<br>transcription regulator	RNA polymerase I transcription factor complex
	superoxide metabolism	superoxide dismutase activity<br>copper, zinc superoxide dismutase activity<br>antioxidant activity<br>oxidoreductase activity<br>metal ion binding	
Single females	microtubule-based process	microtubule motor activity<br>calcium ion binding	microtubule associated complex
		motor activity<br>ATP binding	myosin
		kinase activity<br>transferase activity<br>transferase activity transferring phosphorus-containing groups	
Paired males	RNA-dependent DNA replication	RNA binding<br>RNA-directed DNA polymerase activity	

Table depicting the GO categories of the highly expressed genes which were found in the intersection of our results with the results published by Fitzpatrick and colleagues (2007). Each row in the table represents all the GOs (for each category) for a single gene present in the common results between the two datasets.

Figure - Common genes between datasets

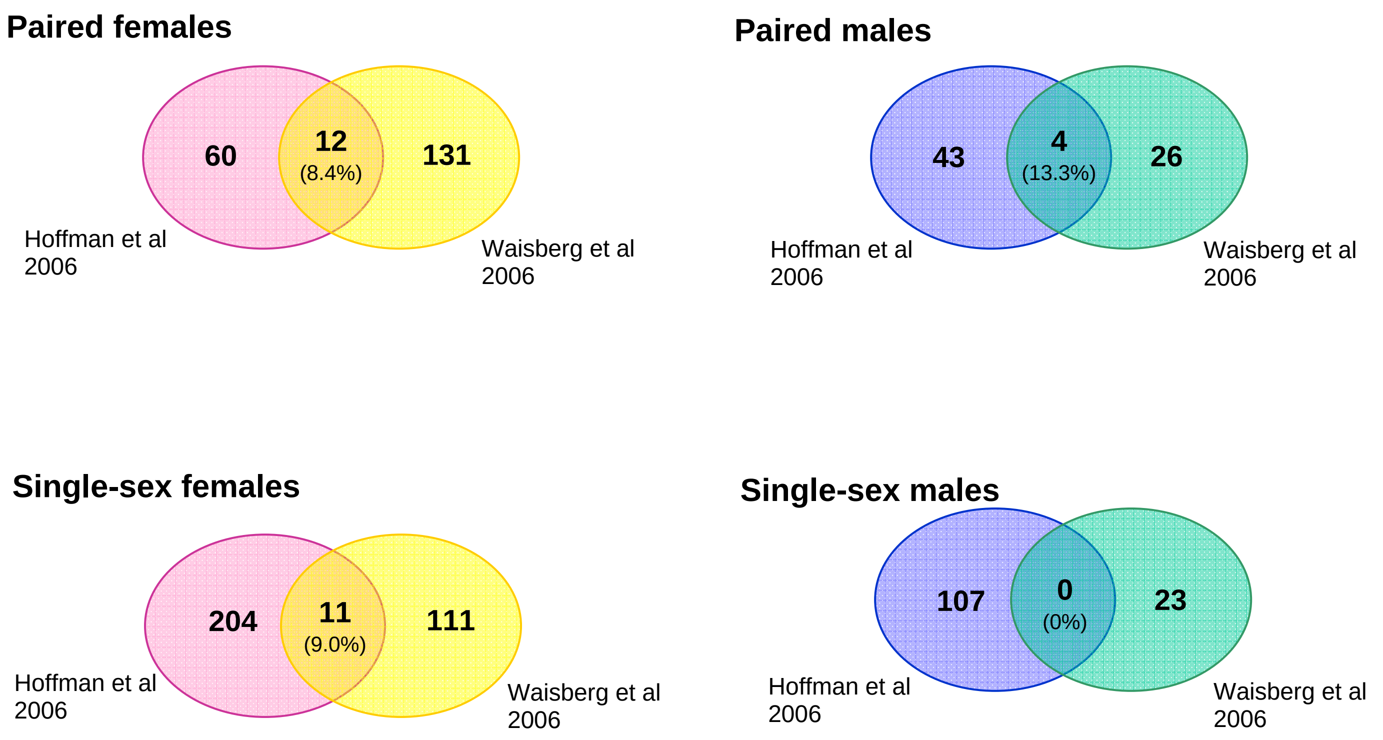

Venn diagrams representing the number of genes and percentage of common genes between our dataset and Fitzpatrick and colleagues (2007) dataset. The percentages were calculated by dividing the number of genes in the intersection by the total number of genes in our dataset.
